# Pathophysiology of Atherosclerotic Plaque Development-Contemporary Experience and New Directions in Research

**DOI:** 10.3390/ijms22073513

**Published:** 2021-03-29

**Authors:** Michal Kowara, Agnieszka Cudnoch-Jedrzejewska

**Affiliations:** Laboratory of Centre for Preclinical Research, Department of Experimental and Clinical Physiology, Medical University of Warsaw, Banacha 1b, 02-097 Warsaw, Poland; michal.kowara@wum.edu.pl

**Keywords:** atherosclerotic plaque, atherogenesis, epigenetics, genetics, macrophage, inflammation, non coding RNA, investigations, future perspectives

## Abstract

Atherosclerotic plaque is the pathophysiological basis of important and life-threatening diseases such as myocardial infarction. Although key aspects of the process of atherosclerotic plaque development and progression such as local inflammation, LDL oxidation, macrophage activation, and necrotic core formation have already been discovered, many molecular mechanisms affecting this process are still to be revealed. This minireview aims to describe the current directions in research on atherogenesis and to summarize selected studies published in recent years—in particular, studies on novel cellular pathways, epigenetic regulations, the influence of hemodynamic parameters, as well as tissue and microorganism (microbiome) influence on atherosclerotic plaque development. Finally, some new and interesting ideas are proposed (immune cellular heterogeneity, non-coding RNAs, and immunometabolism) which will hopefully bring new discoveries in this area of investigation.

## 1. Introduction

Atherosclerotic plaque development and rupture leads to life-threatening diseases such as myocardial infarction, which is one of the main causes of mortality worldwide [1]. It is generally accepted that the interplay between endothelial cell activation, monocyte infiltration, low-density lipoproteins (LDL) particle internalization by macrophages, foam cell generation, local inflammation, and extracellular matrix modification constitutes the basis of atherosclerotic plaque pathogenesis [2]. Immune reactions driven by different cytokines and lipid mediators, as well as local hemodynamic forces affecting endothelial cells, regulate plaque development [3,4]. Nevertheless, many aspects of the pathophysiological mechanisms and molecular pathways leading to atherosclerotic plaque initiation and progression still need to be discovered. Some trends can be noticed among numerous different scientific investigations on atherosclerotic plaque published in recent years. This minireview aims to describe these trends.

## 2. Basic Knowledge

**Figure 1:**
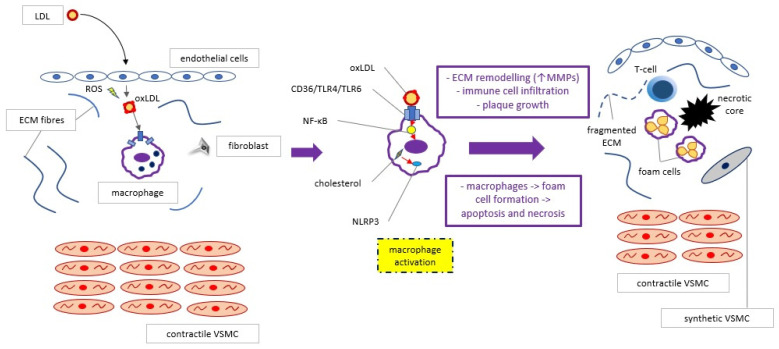
The essential stages of the process of atherosclerotic plaque initiation and progression. ROS—reactive oxygen species, ECM—extracellular matrix, VSMC—vascular smooth muscle cell, CD36—cluster of differentiation 36, TLR4/TLR6—Toll-like receptor 4/6, NLRP3—Nod-like receptor protein 3, NF-κB—nuclear factor kappa B, MMPs—matrix metalloproteinases.

The current view on the process of atherosclerotic plaque progression has been widely described in reviews by Webers, Shami, Basatemur, and Yu, and is illustrated in Figure 1 [4,5,6,7].

**Figure 2:**
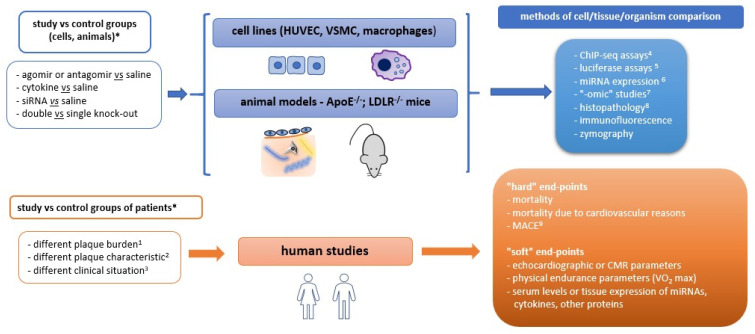
Methodological approaches in studies on atherosclerotic plaque development and progression: *—example studies; 1—assessed by computed tomography coronary angiography (CTCA), 2—assessed by intravascular ultrasound (IVUS) or positron emission tomography (PET); 3—for instance: chronic vs. acute coronary syndrome patients; 4—chromatin state assessment (ChIP—chromatin immunoprecipitation); 5—promoter and transcription factor assessment; 6—by microarray, next-generation sequencing (NGS) and quantitative real-time polymerase chain reaction (qRT-PCR); 7—systemic studies, i.e., genomic (genome-wide association study—GWAS), proteomic, transcriptomic, metabolomic; 8—hematoxylin-eosin staining, immunohistochemistry; 9—major adverse cardiovascular events (MACE), i.e., myocardial infarction, stroke, need for urgent revascularization. Other abbreviations: siRNA—small interfering RNA, HUVEC—human umbilical vein endothelial cell, miRNA—microRNA, CMR—cardiac magnetic resonance imaging, ApoE—apolipoprotein E, LDLR—low-density lipoprotein receptor, VO_2_ max—maximum rate of oxygen consumption.

Briefly, disturbed laminar flow causes endothelial dysfunction, which enables LDL particles to cross the endothelial layer and accumulate within the intima. After modification (i.e., oxidation caused by myeloperoxidase derived from inflammatory cells), the LDL particles activate CD36 receptors on macrophages, which create complexes with TLR4 and TLR6 receptors and transduce an intracellular signal leading to NF-κB activation and chemokine expression. The chemokines (especially chemokine -CC-motif ligands: CCL2, CCL5 and chemokine -CXC- motif ligand 4, i.e. CXCL4) stimulate immune cell infiltration into the intima. Atherosclerotic plaque development is initiated. Macrophages phagocytize more and more LDL particles and evolve into foam cells, which are susceptible to apoptosis and, finally, necrosis (due to impaired efferocytosis, i.e., debris cleavage, mediated by tyrosine-protein kinase Mer, i.e. MertK). Smooth muscle cells switch from a contractile into a synthetic phenotype and synthesize extracellular matrix components (i.e., proteoglycans, collagens), which construct the plaque scaffold and fibrous cap covering, leading to an increase in the entire plaque size. Moreover, cholesterol crystals appearing simultaneously within the macrophages induce interleukin-1-beta (IL-1β) synthesis via NLRP3 inflammasome, enhancing the inflammatory state within the plaque. Other immune cells infiltrating the plaque —neutrophils, T- and B-lymphocytes, and dendritic cells—orchestrate reactions that might lead to plaque destabilization and rupture, which manifests clinically as acute coronary syndrome. Nevertheless, many aspects of the aforementioned processes are still unknown and current investigations aim to explain them more deeply. The next sections present the directions of these studies, whereas Figure 2 illustrates a summary of the methodological approaches.

## 3. Exploration of Regulatory Proteins and Pathways

In recent years, many new discoveries of regulatory pathways have been published. The first group of studies concentrated upon processes involving intimal macrophages and endothelial cells. Taking into consideration that there are different subsets of macrophages within atherosclerotic plaque, a study on genetically modified ApoE^-/-^ mice showed that the absence of chemokine-like receptor ChemR23 caused altered macrophage polarization towards anti-inflammatory M2 phenotype leading to the inhibition of plaque formation and progression [8,9]. Indeed, it must be emphasized that macrophages are crucial cells in the development and progression of atherosclerotic plaque and their different functions and activities are investigated as a hope for discoveries of new antiatherogenic mechanisms [10]. It is already known that there are at least two different subtypes of macrophages—M1, which presents pro-inflammatory and pro-atherogenic activities, and M2, which activities are anti-inflammatory and anti-atherogenic. The pathways leading to macrophage polarization towards M1 or M2 phenotype are still under exploration. As a result, it has been revealed that proinflammatory stimuli such as bacterial lipopolysaccharide (LPS) or interferon-γ induce M1 macrophages, whereas anti-inflammatory cytokines such as interleukins - IL-4, IL-13, IL-10 or transforming growth factor beta (TGF-β) induce the M2 macrophage subpopulation. The M1/M2 macrophage balance within the plaque depends also on transcription factors such as interferon-regulatory factors (IRF)—IRF3 promotes macrophage polarization towards the M2 phenotype (by phosphoinositide kinase 3 (PI3K)/ protein kinase B (Akt) signaling), whereas M5 induces macrophage polarization towards the M1 phenotype [11]. Numerous studies demonstrated that changing the balance between M1 and M2 macrophages in favor of M2 is atheroprotective, as has been shown for ChemR23 inhibition. Similarly, IRF5 silencing via siRNA in ApoE^-/-^ mice results in decreased M1 macrophages and accelerated wound healing after myocardial infarction, whereas transgenic absence of *IRF5* gene (i.e., ApoE^-/-^ IRF5^-/-^ double knock-out) results in decreased necrotic core formation [12,13]. However, the macrophages are not only “coordinating cells” for plaque development, because they also actively participate in this process. As mentioned above, macrophages within the plaque evolve into lipid-laden foam cells and finally undergo the processes of cellular death, apoptosis and necrosis, forming necrotic cores. The latest investigations have shown that apoptosis and necrosis are not the sole processes important for the atherosclerotic plaque development, but alternative processes of cellular death such as necroptosis or autophagy also play a role. Necroptosis is a specific type of cellular death similar to necrosis. The crucial difference between these two processes is that necrosis is chaotic, whereas necroptosis is programmed and guided by signaling pathways. It is initiated by tumor necrosis factor (TNF) or TLR receptors and mediated by mixed-lineage kinase domain-like (MLKL) proteins, which homooligomerize and construct an assembly that facilitates cellular membrane rupture. The silencing of MLKL gene expression results in a decrease in necrotic core size and a decrease in dead cells with a concomitant increase in foam cells due to altered lipid trafficking within macrophages [14]. Other important mechanism of cellular death within atherosclerotic lesion is autophagy, which is a ‘housekeeping’ subcellular process enabling lysosome-mediated hydrolysis of damaged cellular organelles mediated by autophagy-related (ATG) genes. Different studies have demonstrated a positive, i.e., inhibitory role of autophagy in atherosclerotic plaque development. For instance, autophagy promotion through transcription factor EB (TFEB) augmentation or AMP-activated protein kinase (AMPK) / mechanistic target of rapamycin (mTOR) activation (e.g., by cordycepin, an adenosine derivate) results in the inhibition of atherogenesis [15,16,17]. Moreover, new discoveries in the field of already known factors have brought additional data about complex role of macrophages during atherogenesis. As an example, a multifunctional cell-surface protein, CD13, which stimulates inflammatory processes within the macrophage, also plays a role in atherosclerotic plaque development. However, knock out of the CD13 gene resulted in increased atherosclerotic lesions due to augmented nitric oxide and reactive oxygen species synthesis [18]. Such studies highlighted the complex role of certain proteins and explained why simple blockades of a proinflammatory factor often do not result in spectacular attenuation or regression of atherosclerotic plaque. However, not only macrophages contribute to the plaque development. The first line which must be crossed by LDL particles or inflammatory cells is the endothelial layer within the intima. The process of LDL transcytosis across the endothelial cell layer is mediated by protein, caveolin-1 (Cav-1), which enables vesicle formation. It has been discovered that genetic silencing of Cav-1 results in increased autophagy and decreased atherogenesis due to decreased LDL accumulation within the plaque [19,20]. The endothelium, as a barrier which separates the blood from the blood vessel, exposes a numerous adhesive molecules—selectins, integrins and immunoglobulin-like particles, which allow for immune cells translocation (diapedesis), and their inhibition causes a decrease in cell infiltration within the atherosclerotic plaque [21]. Importantly, new discoveries demonstrate that the endothelial cells do not compose passive barrier, but rather an active regulator of the processes within the vessel (and the plaque). The study on ApoE^-/-^ mice with endothelially specific knock-out or overexpression of Foxp1 demonstrated an association between endothelial Foxp1 expression and the advancement of atherosclerotic plaque development [22]. However, the studies concentrated not only on macrophages and endothelial cells, but also on other immune cells. The role of neutrophils, eosinophils and cytotoxic T lymphocytes (especially CD8+) as pro-atherogenetic cells and regulatory T lymphocytes (CD 4 positive, CD25 positive) as anti-atherogenic cells has been already acknowledged [23,24,25]. Nevertheless, the role of many other immune cells, such as Th17 (a subpopulation of helper T lymphocytes) or B lymphocytes, responsible for humoral immune response through antibodies generation during the plaque development, is still fairy known. Regarding B lymphocytes, an investigation of atherosclerotic-prone mice with an attenuated B lymphocyte-induced maturation protein 1 (Blimp-1) gene responsible for B-cell maturation revealed an important pro-atherogenic role of these cells and IgG antibodies [26]. Other investigations highlighted the role of immunoglobulins and their receptors, i.e., IgE and FcεR1, in macrophage polarization towards proinflammatory M1 phenotype, and also highlighted the role of FcγRIIb localized on dendritic cells in sex-dependent differences in the mouse model of atherogenesis [27,28]. In contrast, studies on Th17 lymphocytes provided conflicting results, although an investigation of ApoE^-/-^ mice has revealed increased Th17 lymphocytes among splenocytes in animals with ruptured plaque (induced by LPS and phenylephrine) and increased IL-17 levels within the plaque itself, suggesting a role of this subset of lymphocytes in the plaque rupture [29,30]. Last but not least, not only the cells participate in atherosclerotic plaque progression. Indeed, the plaque development occurs within the scaffold composed of collagen fibers, elastin, and proteoglycans, which form a highly advanced structure in the vascular wall called the extracellular matrix. Thus, atherosclerotic plaque development is associated with alteration of this scaffold. It is known that the plaque progression and destabilization is associated with collagen fiber fragmentation. A study upon human subjects (Cardiovascular Health Study cohort from the USA) demonstrated a relationship between serum markers of collagen turnover (e.g., CITP, telopeptide of collagen type I and cardiovascular events in the elderly) [31]. In addition, other new studies concentrated on extracellular proteins which regulate the extracellular matrix in a more sophisticated way. One example is netrin-1, a protein belonging to the family of axonal migration regulators called netrins. A translational study on netrin-1 (normal and mutated protein—mutation site obtained from the whole genome sequencing of a 30-year-old male patient hospitalized for myocardial infarction) revealed that this protein inhibits monocyte adhesion to endothelial cells and decreases the expression of proinflammatory cytokines (IL-6, CCL-2) and the expression of cell adhesion molecules (ICAM-1) [32]. The inverse correlation between the netrin-1 serum level and the atherosclerotic plaque burden assessed by computed tomography has been observed in a human clinical study [33]. In summary, experiments aiming to discover new regulatory pathways and proteins involved in atherosclerotic plaque development are mainly based upon knock-out genes and antisense oligonucleotides (ASO) in animal models (Figure 1). The typical animals used in such investigations are ApoE^-/-^ or LDLR^-/-^ mice—the control groups were wild type and the study groups had single or double mutations (single or double knock-out organisms). It is known, however, that atherogenesis in mice differs from the corresponding process in humans (e.g., due to a different macrophage phenotype). Therefore, the results from studies on mice should be interpreted with caution. On the other hand, promising findings from mouse experiments are used as a basis for human clinical studies, as mentioned above in reference to netrin-1.

## 4. Exploration of Epigenetic Regulations and Genetic Patterns

Atherosclerosis, although not a genetic disease, depends not only on environmental but also on some congenital factors. Therefore, many studies aimed to find correlations between some genetic polymorphisms (e.g., single-nucleotide polymorphisms—SNPs) and risk of cardiovascular events related to atherosclerotic plaque rupture have already been conducted. For instance, a specific lymphotoxin-alpha genetic variant (on 6p21 locus) may cause a 1.78-fold increase in the risk of myocardial infarction [34]. Nowadays, owing to the new methods implemented in genetic and epigenetic studies (such as next generation sequencing), as well as advanced bioinformatic tools, new discoveries in the field of atherosclerotic plaque development have been made possible. A large-scale genetic study on about 130,000 individuals performed by Eales et al. revealed that carriers of haplogroup IA of the male-specific region of the Y chromosome (MSY) are more susceptible to coronary artery disease. Moreover, sites of chromatin immunoprecipitation sequencing (ChIP-seq) obtained from the Encyclopedia of DNA elements (ENCODE) integrated with known epigenomes collected by The Roadmap Epigenomics Consortium has revealed that haplotype 1A carriers demonstrate significantly increased (by about 50–150%) active chromatin states in the MSY region [35,36]. A greater number of active chromatin states reflects an increased intensity of gene transcription. Additional transcriptome analysis and RNA silencing on macrophages demonstrated that genes differentially expressed in haplotype 1A carriers are under the partial control of the *UTY* gene, which encodes histone demethylase [35]. The genome-wide association studies and genome-wide studies for the DMR (differentially methylated regions) make it possible to find associations between certain gene alleles and coronary artery disease risk as well as between methylation pattern (hyper- or hypomethylation) and atherosclerotic lesion occurrence [37,38]. As mentioned above, the expression of certain genes relies on chromatin status, which is affected by histone methylation or acetylation. The specific pattern of histone methylation, H3K9me2, in vascular smooth muscle cells (VSMC) attenuates the induction of matrix metalloproteinases (MMP3, MMP9, MMP12). Immunofluorescence images have shown that this pattern of methylation is reduced in atherosclerotic lesions in the mouse model. In addition, ChIP analysis in primary human VSMC has revealed that treatment with an inhibitor of the H3K9 methyltransferase resulted in the increased binding of the cJUN (a NF-κB subunit) and p65 (an activator protein-1, i.e. AP-1 subunit) transcription factors to the *MMP3* and *IL6* promoters, respectively. This study provided evidence that the histone methylation state precisely modulates the expression of proinflammatory cytokines and the extracellular matrix hydrolyzing enzymes during atherogenesis [39]. Therefore, many contemporary studies upon epigenetic regulation of atherosclerotic plaque development concentrate not upon known and specific proinflammatory transcription factors (such as NF-κB), but upon peculiarities of general mechanisms guiding epigenetic regulation (such as histone code). In addition, the process of atherogenesis is also investigated in reference to more general transcription factors such as sonic hedgehog (SHH) [40]. Another field of epigenetic studies is the world of microRNA (miRNA). These small particles transcribed from non-coding DNA fragments form an active complex with proteins (RNA-inducing silencing complex—RISC) which cause the degradation of mRNA transcripts complementary to the miRNA. Therefore, the expression of different genes is regulated on the level of mRNA. As an miRNA particle (miR) can silence the expression of different genes and a particular mRNA can be silenced by different miRNA, this mechanism of regulation comprises a huge network of possible interactions [41]. In recent years, many papers describing new regulatory pathways based upon microRNA particles in atherosclerotic plaque development have been published [42]. For example, miR-23a-5p has been described as an ATP-binding cassettes ABCA1/G1 transporter expression negative regulator and its inhibitor (antagomir) causes a decrease in foam cell formation and an enhancement of reverse cholesterol transport [43]. Another study has shown that the peroxisome proliferator-activated receptor gamma (PPARγ) coactivator, i.e. PCG1α (a regulator of cellular energy homeostasis), together with nuclear respiratory factor 1 (NRF1) transcription factor activates the synthesis of miR-378a, a negative regulator of the *insulin-growth factor 1* (*IGF1)* and *TLR8* genes. The fact that PCG1α is decreased in the VSMC of atherosclerotic vessels highlights the role of the PCG1α/NRF1/miR-378a axis in atheroprotection [44]. Moreover, a new technique in genetic and epigenetic studies has appeared in recent years—gene editing, a procedure in which certain fragments of genes or non-coding sequences can be altered with the use of Clustered Regularly Interspaced Short Palindromic Repeats (CRISPR) and CRISPR-associated protein 9 (Cas9) systems built up with guide RNA [45,46]. This method opens up new horizons in the area of precise genomic and epigenomic research.

## 5. Exploration of the Influence of Biophysical Factors upon Atherogenesis

The aspect of the influence of different biophysical factors (i.e., hemodynamical forces, blood flow) on atherosclerotic plaque development and progression is particularly important from the point of view of human physiology. Experiments have compared cellular reactions under different biophysical conditions. He et al. showed that human umbilical vein endothelial cells (HUVEC) exposed to atheroprotective pulsatile shear stress (12 ± 4 dyn/cm^2^) demonstrate a different epigenetic pattern (i.e., H3K27 acetylation enrichment in *ITPR3* (*inositol 1,4,5-trisphosphate receptor*) promoter region, making it more accessible for Krüppel-like factor 4, i.e. KLF4) than cells exposed to proatherogenic oscillatory shear stress (1 ± 4 dyn/cm^2^). Therefore, pulsatile shear stress induces increased NO bioavailability due to ITPR3 mediated endothelial nitric oxide synthase (eNOS) activation in HUVEC, which makes their phenotype more antiatherogenic [47]. Laminar blood flow, unlike oscillatory flow, stabilizes the endothelial glycocalyx layer on the cell surface through increased hyaluronan synthesis and glucosamine bioavailability mediated by Krüppel-like factor 2 (KLF2) [48]. A test with acetylcholine performed on patients with previous myocardial infarction has shown that advanced atherosclerotic lesions with a thickened media layer measured by OCT (optical coherence tomography) are characterized by an attenuated contractile response [49]. However, it is still unknown as to how mechanical forces modulate the intracellular pathways. One proposed explanation is that ion transport across the cellular membrane is mediated via mechanosensitive Piezo ion channels [50].

## 6. Exploration of Tissues and Microorganisms Affecting Atherogenesis

Studies on atherosclerotic plaque development concentrate also on tissues and microorganisms that might influence this process. One concept is the phenomenon of endothelial-to-mesenchymal cell transition (EndMT), which might explain the origin of plaque-associated mesenchymal cells [51]. According to this concept, endothelial cells might convert into mesenchymal cells which express both endothelial (e.g., platelet endothelial cell adhesion molecule-1, i.e. PECAM-1) and mesenchymal (e.g., alpha smooth muscle actin, i.e. α-SMA) markers and contribute directly to plaque progression. This process is supposed to be driven by a cytokine TGF-β. Apart from that, a new tissue which is considered to be a player in the process of atherogenesis is perivascular adipose tissue (PVAT), which generates paracrine factors affecting vessel homeostasis [52]. For instance, ApoE^-/-^ mice with PPARγ knock-out specific to brown adipose tissue (and an altered PVAT excretory profile) demonstrated a significantly increased number of atherosclerotic lesions [53]. Finally, a new important field of investigation is the influence of the gut microbiota on atherogenesis. An animal study showed that proinflammatory microbiota from Caspase^-/-^ mice transplanted into atherosclerosis-prone ApoE^-/-^ mice intensifies systemic inflammation and augments atherosclerosis [54]. Similar effects of different microbiomes on atherogenesis were also observed in humans. For instance, a study on a subsample of 112 participants from the Bogalusa Heart Study revealed that eight taxa were significantly associated with increased cardiovascular risk, i.e., *Alloprevotella, Prevotella 7, Paraprevotella, Tyzzerella, Tyzzerella 4, Megamonas, Catenibacterium,* and *Enterobacter* [55]. It is supposed that the choline metabolite, trimethylamine N-oxide (TMAO), is the main microbiota-derived proatherogenic factor [56]. However, the exact mechanisms of the influence of the microbiome on atherogenesis are still to be elucidated [57]. Last but not least, it has been discovered that many cells in human organism communicate with each other through extracellular vesicles and it also refers to the communication between endothelial and immune cells [58]. The role of extracellular vesicles as biomarkers and possible causative factors in cardiovascular diseases has also been discussed [59].

## 7. New Inspiring Ideas for Further Investigation

**Figure 3:**
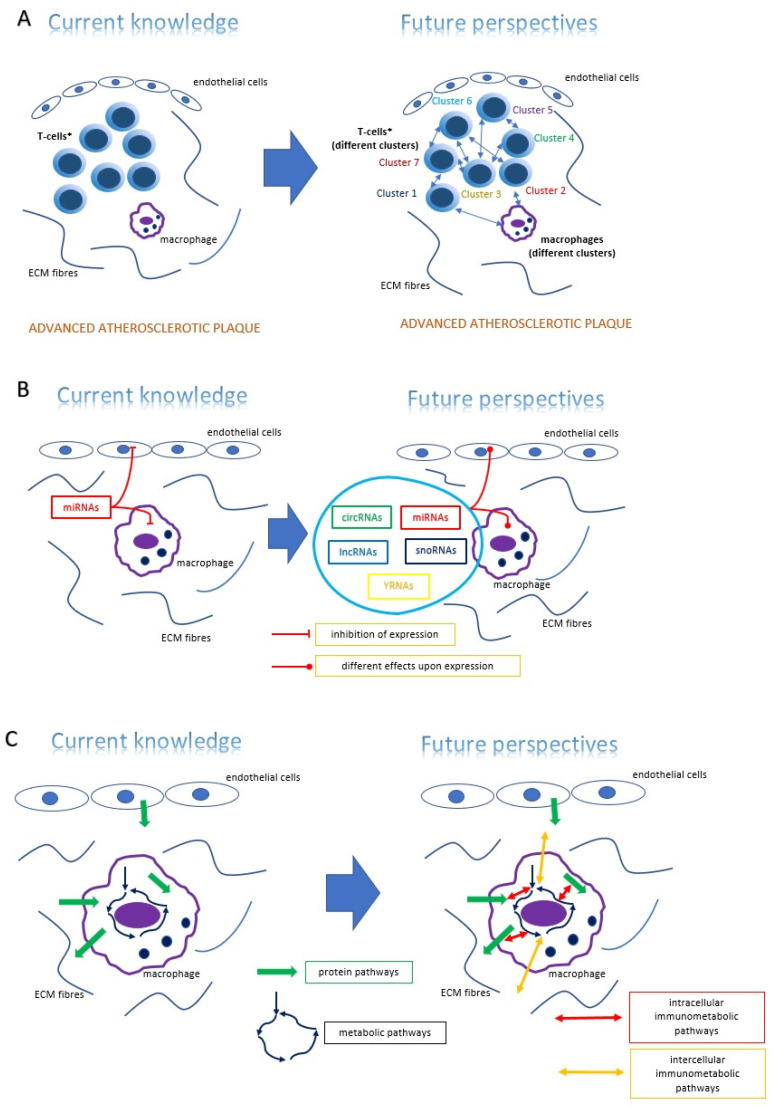
Insight into the perspectives of future research on atherosclerotic plaque—three main proposed directions of exploration: (**A**)—heterogeneity of immune cells, (**B**)—non-coding RNA influence, (**C**)—immunometabolism.

Although there is an awareness of the presence of different immune cells within the plaque that interfere with the process of atherogenesis, the heterogeneity and different patterns of functionality of these cells have not yet been explored. A new technique called single cell RNA sequencing (scRNAseq) makes transcriptome examination of every single cell within the investigated tissue possible due to selective sequencing (by the NGS method) of cell barcodes called unique molecular identifiers (UMI). Therefore, different cellular clusters characterized by certain UMI and therefore certain transcription profiles can be marked [60]. An interesting study on ApoE^-/-^ mice revealed 17 immune cell clusters within the plaque and showed that atherosclerotic plaque regression (obtained by the administration of apolipoprotein B antisense oligonucleotide) coincides with alterations of the transcription profile within regulatory T-cell (Treg) clusters, i.e., decreased *neuropilin-1* (*NRP1*)—*thymic marker; increased enolase 1* (*ENO1*), *ribulose-5-phosphate-3-epimerase* (*RPE*), and *carnitine palmitoyltransferase 1A* (*CTP1A*)—metabolism associated genes [61]. Questions include whether these subsets of immune cells differ significantly from each other from functional point of view? Is there a subset that significantly affects atherogenesis? These issues are still to be determined. A second area of hopeful investigation for new discoveries is the world of non-coding RNA. To date, many microRNA particles have been identified, which makes discoveries of new molecular pathways and feedback loops in atherogenesis possible. It is known how a microRNA particle affects the expression of other genes and proteins. However, not only microRNAs play a role in this process. The genome possesses many fragments encoding so called “non-coding RNAs”, which are longer and form different structures. This group includes YRNAs, small nucleolar RNA (snoRNAs), circular RNA (circRNA), and finally long non-coding RNA (lncRNAs). Although the function and biology of all of these particles is fairly well understood, there is some evidence of their participation in cardiovascular pathophysiological processes such as atherogenesis. For instance, lncRNA called MANTIS inhibits pro-inflammatory *ICAM-1* expression in endothelial cells, probably by affecting chromatin remodeling [62]. A study by Li et al. enlightened the complexity of circRNA physiology by showing that the circRNA_000203 circular particle possesses sequences enabling miR-126-5p and miR-140-3p binding. These miRNA particles, while being bound by circRNA_000203, are unable to inhibit *GATA-4* expression, which results in increased GATA-4 target gene expression leading to cardiomyocyte hypertrophy [63]. However, the world of non-coding RNA still remains undiscovered in many aspects. Are these newly discovered particles significant players which integrate and orchestrate different molecular reactions in atherosclerotic plaque progression, or only additional factors, the role of which in plaque progression is auxiliary? Will ncRNA particles become biomarkers or therapeutic agents in future cardiovascular medicine? These questions remain open. A third area of hopeful investigation is immunometabolism—a return to studies on biochemical reactions involved in metabolic reactions within a single cell (glycolysis, oxidative phosphorylation, fatty acid oxidation) with the focus on interactions between metabolites and molecular pathways mediated by proteins, as well as the paracrine effects of the metabolites on surrounding cells. Although the Warburg effect (“aerobic glycolysis”, i.e., a preference of a single cell for metabolism towards glycolysis rather than oxidative phosphorylation even under the condition of sufficient oxygen supply) has been known since the 1920s, its biological role still needs to be explained. It is supposed that LPS-mediated activation of macrophages, which promotes “aerobic glycolysis” through upregulation of key kinases involved in glycolysis, is the means to promote a maximum inflammatory response of the cell and a quick termination of this response at a later time [64]. Metabolism is a dynamic process and interaction of different metabolites with entire cellular machinery will vary according to the cell activity. Therefore, new molecular investigations linking metabolism with signaling pathways (mediated by proteins) might also be adjusted for kinetics of metabolic reactions. This opens up a new field of research in which biomathematics plays an important role. The perspectives of research in the abovementioned areas are briefly illustrated in Figure 3.

In addition, there is also hope in well-investigated areas of research on atherosclerotic plaque which may result in interesting discoveries. For instance, bromodomain and extraterminal-containing protein family (BETs), which establish certain transcriptional programs in cells through binding to specifically marked residues on histone tails or, for instance, neutrophil extracellular traps (NETs), which are web-like extrusions of genetic materials derived from neutrophils that are probably involved in the regulation of important processes such as atherosclerotic plaque development, rupture and thrombosis [65,66,67]. Will the discoveries such as BETs or NETs change our perspective on the processes of gene expression and intercellular reactions within the plaque? Currently, it is difficult to respond to this question, but such discoveries certainly complement the knowledge about atherogenesis and open up new areas of basic research. Moreover, new technological advances in physics and informatics will also affect investigations in cardiovascular medicine. Shall we be able to investigate hundreds of reactions simultaneously within the plaque? Will the relations between elementary particles investigated today by theoretical physicists turn out to be important also in cardiovascular pathophysiology? These issues are rather considerations for more distant future. Last but not least, some classic ideas such as the role of reactive oxygen species (ROS) are still debated. Although the role of ROS in atherogenesis is generally known (and considered proatherogenic), many specific details about their generation and activity are still being discovered and constitute the basis for new hypotheses [68,69]. However, processes involving ROS are difficult to determined and calculate, and therefore it is easy to either overestimate or underestimate the role of these particles in the process of atherosclerotic plaque development.

## 8. Conclusions

Investigations on atherosclerotic plaque development still open up new areas of research and lead to the discovery of novel pathways involved in this complex process. In the past, techniques such as transgenic organism generation (e.g., ApoE^-/-^, LDLR^-/-^, or double knock-out mice), RNA silencing, polymerase chain reactions, microarrays, chromatin immunoprecipitation assays made possible many discoveries in the field of atherogenesis. Today, due to advances in methodology in recent years (large-throughput “-omics” studies, next generation sequencing, bioinformatic tools and data libraries, the CRISPR-Cas9 gene editing system, single cell RNA sequencing) new pathways and regulatory mechanisms are still being revealed. New fields of investigations such as the exploration of cellular heterogeneity within the plaque, non-coding RNA functions, and immunometabolism are a hopeful basis for new promising discoveries.

## Data Availability

Not applicable.
